# Retrospective study of immunization errors reported in an online Information System*

**DOI:** 10.1590/1518-8345.3343.3303

**Published:** 2020-06-19

**Authors:** Tânia Cristina Barboza, Rafael Alves Guimarães, Fernanda Raphael Escobar Gimenes, Ana Elisa Bauer de Camargo Silva

**Affiliations:** 1 Secretaria de Estado de Saúde de Goiás, Superintendência de Vigilância em Saúde, Goiânia, GO, Brazil.; 2 Universidade Federal de Goiás, Faculdade de Enfermagem, Goiânia, GO, Brazil.; 3 Universidade de São Paulo, Escola de Enfermagem de Ribeirão Preto, PAHO/WHO Collaborating Centre for Nursing Research Development, Ribeirão Preto, SP, Brazil.; 4 Universidade Federal de Goiás, Faculdade de Enfermagem, Goiânia, GO, Brazil.

**Keywords:** Vaccination, Immunization, Patient Safety, Electronic Health Records, Adverse Effects, Drug-Related Side Effects and Adverse Reactions, Vacinação, Imunização, Segurança do Paciente, Registros Eletrônicos de Saúde, Efeitos Adversos, Efeitos Colaterais e Reações Adversas Relacionados a Medicamentos, Vacunación, Inmunización, Seguridad del Paciente, Registros Electrónicos de Salud, Efectos Adversos, Efectos Colaterales y Reacciones Adversas Relacionados con Medicamentos

## Abstract

**Objective:**

to analyze the immunization errors reported in an online Information System.

**Method:**

retrospective study conducted with data from the Adverse Event Following Immunization Surveillance Information System. Immunization errors were analyzed with respect to demographic characteristics and the vaccination process. Frequencies and error incidence rates have been calculated. Binomial and chi-square tests were used to verify differences in the proportions of the variables.

**Results:**

501 errors were analyzed, the majority involving routine doses (92.6%), without Adverse Event Following Immunization (90.6%) and in children under five years old (55.7%). The most frequent types of errors were inadequacy in the indication of the immunobiological (26.9%), inadequate interval between doses (18.2%) and error in the administration technique (14.2%). The overall error incidence rate was 4.05/100,000 doses applied; the highest incidences of routine vaccines were for human rabies vaccine, human papillomavirus and triple viral; the incidence rate of errors with Adverse Events Following Immunization was 0.45/100,000 doses applied.

**Conclusion:**

it was found that immunization errors are a reality to be faced by the health systems, but they are amenable to prevention through interventions such as the adoption of protocols, checklists and permanent education in health.

## Abstract (pt)

**Objetivo:**

analisar os erros de imunização notificados em um Sistema de Informação on-line.

**Método:**

estudo retrospectivo realizado com dados do Sistema de Informação da Vigilância de Eventos Adversos Pós-Vacinação. Os erros de imunização foram analisados com relação às características demográficas e ao processo de vacinação. Foram calculadas frequências e taxas de incidência dos erros. Os testes binomial e qui-quadrado foram utilizados para verificar diferenças nas proporções das variáveis.

**Resultados:**

foram analisados 501 erros, a maioria envolvendo doses de rotina (92,6%), sem Evento Adverso Pós-Vacinação (90,6%) e em crianças menores de cinco anos (55,7%). Os tipos de erros mais frequentes foram inadequação na indicação do imunobiológico (26,9%), intervalo inadequado entre doses (18,2%) e erro na técnica de administração (14,2%). A taxa de incidência global de erros foi de 4,05/100.000 doses aplicadas; as maiores incidências das vacinas de rotina foram para vacina antirrábica humana, papiloma vírus humano e tríplice viral; a taxa de incidência de erros com Eventos Adversos Pós-Vacinação foi de 0,45/100.000 doses aplicadas.

**Conclusão:**

verificou-se que erros de imunização são uma realidade a ser enfrentada pelos sistemas de saúde, porém são passíveis de prevenção por meio de intervenções como adoção de protocolos, *checklists* e educação permanente em saúde.

## Abstract (es)

**Objetivo:**

analizar los errores de inmunización reportados en un Sistema de Información en línea.

**Método:**

estudio retrospectivo realizado con datos del Sistema de Información de Vigilancia de Eventos Adversos Posteriores a la Vacunación. Los errores de inmunización se analizaron con respecto a las características demográficas y el proceso de vacunación. Se calcularon las frecuencias y las tasas de incidencia de errores. Se utilizaron pruebas binomiales y de chi-cuadrado para verificar las diferencias en las proporciones de las variables.

**Resultados:**

se analizaron 501 errores, la mayoría con dosis de rutina (92,6%), sin evento adverso posterior a la vacunación (90,6%) y en niños menores de cinco años (55,7%). Los tipos de errores más frecuentes fueron la insuficiencia en la indicación del inmunobiológico (26,9%), el intervalo inadecuado entre las dosis (18,2%) y el error en la técnica de administración (14,2%). La tasa de incidencia de error global fue de 4,05/100.000 dosis aplicadas; las mayores incidencias de las vacunas de rutina fueron para la vacuna contra la rabia humana, el virus del papiloma humano y el triple viral; La tasa de incidencia de errores con los eventos adversos posteriores a la vacunación fue de 0,45/100.000 dosis aplicadas.

**Conclusión:**

se encontró que los errores de inmunización son una realidad que deben enfrentar los sistemas de salud, pero se pueden prevenir mediante intervenciones como la adopción de protocolos, *checklists* y educación permanente en salud.

## Introduction

Immunization is a key component of the efforts made by the World Health Organization (WHO) to achieve the United Nations’ third Sustainable Development objective by 2030, which is to ensure a healthy life, promote well-being to be for all, at all ages, by reducing the infant and maternal mortality rate, from communicable and non-communicable diseases, including ensuring access to safe and quality vaccines^(^ 1 ^)^.

In the last few decades, the number of vaccine-preventable diseases has almost doubled and, to that end, the number of vaccine doses has increased for children and adults^(^ 2 ^)^. With the increase in the number of doses applied, the potential for immunization errors (IE) has also increased globally^(^ 2 ^)^. Furthermore, despite advances in immunization surveillance systems worldwide, IE are often underreported^(^ 2 ^)^. The IE can be conceptualized as any preventable events arising from the inappropriate use of vaccines, which may be related to professional practice and the imprudent use of immunobiologicals, which, outside the norms and appropriate techniques, can lead to negative impacts, such as inadequate immune protection, increased costs for health services, reduction confidence and potential injuries of health system users^(^ 3 ^)^. The IE may cause adverse events following immunization (AEFI) and result in severe harms to the users^(^ 4 ^-^ 6 ^)^.

The IE occur at an alarming frequency^(^ 7 ^)^. Studies conducted in developed countries have found a high rate of IE involved in vaccine administration^(^ 3 ^,^ 8 ^-^ 9 ^)^. Approximately one third of users experience at least one IE^(^ 7 ^)^. In a systematic review carried out in five English-speaking countries, a prevalence of vaccination error of 1.15 *per* 10,000 doses of vaccines was identified^(^ 10 ^)^. In Europe, a study that analyzed data from the AEFI surveillance system showed that of 233,285 vaccination records, in 3.0%, reports of at least one IE were identified, and of these, more than half (59.9% ) resulted in severe outcomes^(^ 8 ^)^. In Canada, a retrospective study of 3,504 reports of AEFI, estimated an IE rate of 0.39 *per* 100,000 applied doses (a.d.)^(^ 11 ^)^. In the United States of America (USA), analysis of the AEFI surveillance system showed that out of 21,843 IE in the country, among which, in 25% of the cases, there was some harm to the user^(^ 3 ^)^.

International literature shows that the most common IE are related to the inappropriate scheme (for example: incomplete vaccination), storage and distribution errors, incorrect vaccine, incorrect doses, incorrect interval/time and administration errors^(^ 3 ^,^ 8 ^-^ 9 ^)^.

Many IE occur at the expense of the complexity of vaccination programs. Brazil offers immunobiologicals to its population, through the National Immunization Program (PNI), one of the most expressive Brazilian public policies, considered one of the most complete and complex vaccination programs in the world, internationally recognized for its excellence^(^ 12 ^-^ 13 ^)^. The PNI has considerably expanded the supply of immunobiologicals in recent years, thus facing new challenges^(^ 12 ^-^ 13 ^)^. Currently, makes available 300 million annual doses of 44 different types of immunobiologicals, including routine vaccines, serums and immunoglobulins in around 34,000 existing vaccination rooms^(^ 14 ^)^.

Since 2009, the PNI has adopted the PNI Information System (IS-PNI) as the AEFI Information System module (IS-AEFI), which, since 2014, has existed in online format, with the objective of favoring better obtaining, description, surveillance and analysis of AEFI and IE data^(^ 12 ^,^ 15 ^-^ 16 ^)^.

In Brazil, studies on the epidemiology of IE are scarce, among them, few have analyzed the types of errors involved in immunization^(^ 6 ^,^ 15 ^,^ 17 ^-^ 18 ^)^. For example, a national study that analyzed 1,622 notifications with closure of IS-AEFI, showed that 9.3% represented IE without AEFI and 0.8% IE with AEFI^(^ 15 ^)^. Another study in Paraná (South Region) showed the occurrence of 604 records of types of AEFI resulting from IE, with frequent hot subcutaneous abscess, cold subcutaneous abscess and suppurated regional lymphadenopathy^(^ 6 ^)^. In Goiânia (State of Goiás, Central-West Region), a study showed that among the 373 AEFI analyzed in children, in 16.1% IE occurred, with the most frequent vaccine administered outside the recommended age, vaccine administered with expiration date, inadequate interval between doses and vaccine applied in the wrong place^(^ 18 ^)^.

There is a gap in the literature on IE, as there are few studies conducted in developing countries, such as Brazil, that show IE magnitude. Most existing studies do not report IE incidence rate stratified by type of immunobiological element. Although the risk to vaccine-related AEFI has received considerable attention in the recent years^(^ 4 ^-^ 6 ^)^, studies on AEFI related to IE have also been underdeveloped.

The analysis of existing data at IS-AEFI can support the planning of public health policies, the strengthening of PNI actions and health services, as well as the reflection on the practice of nursing that works in vaccination rooms, aiming at IE prevention in the country. It is also believed to be able to contribute to fill a gap in the scientific literature in developing countries, such as Brazil, related to the most frequent IE types; the demographic characteristics of the population affected by immunization errors; doses of immunobiologicals; the routes of administration most commonly involved in the events; as well as the identification of the IE incidence rate by type of immunobiological, with and without AEFI.

Thus, this study aimed to analyze the IE notified in Goiás, between 2014 and 2017.

## Method

This is a retrospective study conducted with secondary data from IS-PNI, module IS-AEFI, in the period from August, 2014 (usage date of the on-line of IS-AEFI in the state of Goiás) to December, 2017.

All IE notifications registered in IS-AEFI related to IE, in Goiás, involving routine immunobiologicals, special immunobiologicals, serums and vaccination campaigns were analyzed. Goiás is a state located to the east of the Midwest Region of Brazil, with an area of 340,086 km^2^, an estimated population of 6,921,161 inhabitants in 2018, and a population density of 17.65 inhabitants/km^2^. It is located in the 7^th^ place in the Human Development Index in Brazil. The nominal monthly household income *per capita* is R$ 1,277.00, occupying the 8^th^ place in the country. It has 246 municipalities, the capital of Goiânia being the largest in terms of population^(^ 19 ^)^. Immunobiologicals are available in all municipalities in the State, in vaccination rooms at Basic Health Units, Emergency Care Units, at the Reference Center for Special Immunobiologicals (CRIE) and at some public maternity hospitals. In 2018, the State had 943 public vaccination rooms (active), notifying, offering immunobiologicals in the basic vaccination calendars.

In this study, the data sources used were the online IS-AEFI for collection and analysis of the notified IE and the IS-PNI for obtaining the number of applied doses (a.d.) of each immunobiological from August 2014 to December 2017.

The period of data collection and evaluation of the records occurred from January to February 2018. For data extraction, a standardized data collection form was elaborated, based on the information contained in the IS-AEFI. Initially, the consistency of information from all IE was assessed, and, in the event of more than one IE *per* notification, they were broken down for accounting and analysis.

The following variables of interest, found in the IS-AEFI, were analyzed to characterize the epidemiological profile of the IE in relation to users and characteristics involved in the vaccination process: (i) gender (male or female); (ii) age group stratified into: <1 year, 1-4 years, 5-9 years, 10-19 years, 20-59 years or > 60 years; (iii) race/skin color (white, black/brown, Asian or indigenous); (iv) type of event (IE without AEFI or IE with AEFI); (v) dose (1^st^, 2^nd^, 3^rd^, 4^th^, 1^st^ reinforcement, 2^nd^ reinforcement, unique dose, revaccination or campaign); (vi) route of administration (intramuscular, subcutaneous, oral, intradermal or intravenous); (vii) strategy (routine, special immunobiologicals, serums or vaccination campaign), (viii) type of immunobiological and (ix) types of IE.

The types of IE were categorized into nine categories, as recommended by the Brazilian AEFI Manual^(^ 20 ^)^: (i) prescription or indication errors (that is, vaccine administered before or after the recommended age); (ii) inadequate interval between doses (that is: dose ranges larger or smaller than recommended); (iii) error in the administration technique (that is: track error, topography, hand hygiene, inadequate needle size); (iv) error in the type of immunobiological (that is: incorrect vaccine administration); (v) misuse of diluents or administration of other products (that is, dilution and administration of vaccines with substituted diluents); (vi) error in the evaluation of contraindications or precautions (that is: administration of live vaccines to pregnant or immunocompromised patients); (vii) expired validity (that is: expired vaccine application); (viii) inadequate interval between vaccines (that is Yellow Fever and Triple Viral administered on the same day in children under two years of age); (ix) other IE (that is; errors that are not listed in the above categories, such as registration error).

Data were analyzed in the statistics program, STATA, version 14.0. Initially, a descriptive analysis of the study variables was performed through absolute (n) and relative (%) frequencies. To calculate the overall IE incidence rate (IE without AEFI and IE with AEFI), the number of IE cases reported in the IS-AEFI (numerator) and the number of doses applied (a.d.) were used and registered in the IS-PNI (denominator), using the following formula:

$$\frac{\mathrm{Number}\;\mathrm{of}\;\mathrm{IE}\;\mathrm{cases}\left(\mathrm{EI}\;\mathrm{without}\;\mathrm{AEFI}\;\mathrm{and}\;\mathrm{IE}\;\mathrm{ewith}\;\mathrm{AEFI}\right)}{\mathrm{Total}\;\mathrm{of}\;\mathrm{doses}\;\mathrm{applied}\;\mathrm{in}\;\mathrm{the}\;\mathrm{same}\;\mathrm{period}}\times 100.000$$

In addition, the IE incidence rate without AEFI and with AEFI separately was estimated. All measures of descriptive analysis and incidence rate were followed by the respective 95% confidence intervals (95% CI).

In the following, the differences between the proportions according to demographic characteristics (age group, gender, race/skin color), type of event, dose of immunobiological, route of administration, vaccination strategy and type of error were verified using the binomial test of a sample (for dichotomous nominal variables) or sample chi-square test (for nominal variables with more than two categories or ordinals). A significance level of 5% (p-value <0.05) was adopted to verify the statistically significant variables.

The study was approved by the Research Ethics Committee of Clinics Hospital of the Federal University of Goiás, opinion nº 2.519.065/2018.

## Results

In this study, all 404 notification forms were analyzed and, among them, 72 (17.8%) had a record of two or more IE, totaling 501 IE analyzed in Goiás.

Table 1 summarizes the descriptive analysis of the 501 IE analyzed, according to demographic characteristics, type of event, dose of immunobiological, route of administration and vaccination strategy. A higher proportion of IE was found in females (62.5%; p-value <0.001), in the age group under 1 year (32.7%; p-value <0.001) and in users of black/brown race/skin color (47.7%; p-value <0.001). Also, there was a higher proportion of IE without AEFI than with AEFI (90.6% versus 9.4%; p-value <0.001). Finally, almost half of the IE occurred during the administration of the first vaccine dose (49.7%; p-value <0.001) and the majority involved vaccines administered intramuscularly (58.1%; p-valor < 0.001). Of the total IE, the majority occurred during routine doses (92.6%; p-value <0.001).

**Table 1:** Descriptive analysis of cases of notified immunization errors, according to demographic characteristics, type of error, dose of immunobiological, route of administration and strategy. Goiás State, Brazil, August 2014 to December 2017

Variables	N = 501	%	CI 95%*	p-value
**Gender**				
Male	188	37.5	33.4-41.8	< 0.001^†^
Female	313	62.5	58.2-66.6	
**Age group (years old)**				
< 1	164	32.7	27.8-37.7	< 0.001^‡^
1-4	115	23.0	19.5-26.8	
5-9	23	4.6	3.1-6.8	
10-19	65	13.0	10.3-16.2	
20-59	125	25.0	21.4-28.9	
> 60	9	1.8	0.9-3.4	
**Ethnicity**				
White	63	32.0	25.9-38.7	< 0.001^‡^
Black/brown	94	47.7	40.8-54.7	
Asian	33	16.8	12.2-22.6	
Indigenous	7	3.6	1.7-7.1	
N/A^§^: 304				
**Type of event**				
Immunization error without AEFI^||^	454	90.6	87.7-92.9	< 0.001^†^
Immunization error with AEFI^||^	47	9.4	7.1-12.2	
**Dose**				
1^st^ dose	249	49.7	45.3-54.1	< 0.001^‡^
2^st^ dose	85	17.0	13.9-20.5	
3^st^ dose	28	5.6	3.89-7.96	
4^st^ dose	4	0.8	0.31-2.03	
1^st^ reinforcement	29	5.8	3.06-8.20	
2^st^ reinforcement	22	4.4	2.92-6.56	
Unique	34	6.8	4.89-9.33	
Revaccination	34	6.8	4.89-9.33	
Campaign	16	3.2	1.97-5.12	
**Route of administration**				
Intradermal	15	4.0	2.4-6.5	< 0.001^‡^
Intramuscular	219	58.1	53.0-63.0	
Subcutaneous	80	21.2	17.4-25.6	
Via oral	61	16.2	12.8-20.2	
Intravenous	2	0.5	0.1-1.9	
N/A^§^: 124				
**Strategy**				
Routine	464	92.6	90.0-94.6	< 0.001^‡^
Vaccination campaign	16	3.2	2.0-5.1	
Special immunobiologicals	14	2.8	1.7-4.6	
Serums	7	1.4	0.7-2.9	

Source: Information System of the National Immunization Program (IS-PNI), IS-AEFI module

* CI = 95% confidence interval;

† Binomial test of a sample;

‡ Chi-square test of a sample;

§ N/A = No information;

|| AEFI = Post-Vaccination Adverse Event.

Table 2 shows IE descriptive analysis according to the type of immunobiological. Proportionally, the majority of IE involve the triple viral vaccines (15.4%), yellow fever (12.0%), human papillomavirus (HPV) (10.0%), pentavalent (7.4%) and oral vaccine human rotavirus (OVHR) (7.0%).

**Table 2:** Descriptive analysis of cases of immunization errors, according to the type of immunobiological agent. Goiás State, Brazil, August 2014 to December 2017

Variables	N = 501	%	CI 95%*
**Routine**			
*Bacillus Calmette-Guérin*	12	2.4	1.4-4.1
Adult Double	14	2.8	1.7-4.6
Triple Bacterial	28	5.6	3.9-8.0
Triple Acellular Bacterial Adult	8	1.6	0.8-3.1
Yellow Fever	60	12.0	9.4-15.1
Hepatitis A (pediatric)	7	1.4	0.7-2.9
Hepatitis B	25	5.0	3.4-7.3
Human Papillomavirus	50	10.0	7.6-12.9
Conjugated meningococcal C	31	6.2	4.4-8.6
Pneumococcal 10 valent	18	3.6	2.3-5.6
Pentavalent	37	7.4	5.4-10.0
Triple Viral	77	15.4	12.5-18.8
Tetra Viral	12	2.4	1.4-4.1
Inactivated Polio Vaccine	13	2.6	1.5-4.4
Oral Polio Vaccine	27	5.4	3.7-7.7
Human Rabies Vaccine	10	2.0	1.1-3.6
Oral Human Rotavirus Vaccine	35	7.0	5.1-9.6
**Vaccination campaign**			
Trivalent influenza	16	3.2	2.0-5.1
**Special immunobiologicals**			
Triple Acellular Bacterial Infant	2	0.4	0.1-1.4
Vaccine *Haemophilus influenzae* type b	1	0.2	0.0-1.1
Immunoglobulin anti-hepatitis B	1	0.2	0.0-1.1
Pneumococcal 23 valent	8	1.6	0.8-3.1
Hepatitis A (CRIE^†^)	2	0.4	0.1-1.4
**Serums**			
Scorpion serum	5	1.0	0.4-2.3
Serum against tetanus	1	0.2	0.0-1.1
Botropic serum	1	0.2	0.0-1.1

Source: Information System of the National Immunization Program (IS-PNI), IS-AEFI module

* 95% CI = 95% confidence interval;

† CRIE = Reference Center for Special Immunobiologicals

The most frequent types of errors indicated inadequacy in the indication of the immunobiological (26.9%; 95% CI: 23.2-31.0), inadequate interval between doses (18.2%; 95% CI: 15.0-21.8) and error in the administration technique (14.2%; 95% CI: 11,4-17,5). The prevalences of the other types of IE were: error related to the type of used immunobiological (11.8%; 95% CI: 9.2-14.9), administration error - incorrect use of diluents (8.4%; 95% CI: 6.3-11.1), error in the evaluation of contraindications or prescriptions (7.8%; 95% CI: 5.7-10.5), expired validity (5.4%; 95% CI: 3.7-7.7%), inadequate interval between vaccines (0.6%; 95% CI: 0.2-1.7) and other errors (6.8%; 95% CI: 4.9-9.3%) (data not shown in tables).

Table 3 shows the global incidence rate of IE (per 100,000 a.d.). The overall incidence rate was 4.05 IE/100,000 a.d. Vaccines administered routinely had an incidence rate of 3.70/100,000 a.d. and serums of 547.70 IE/100,000 a.d. The three highest incidence rates for routine vaccines were found for the human rabies vaccine (24.93 IE\/100,000 a.d.), HPV (10.12/100,000 a.d.) and triple viral (8.74/100,000 a.d.).

**Table 3:** Global incidence rate of immunization errors (*per* 100,000 applied doses), according to the vaccination strategy and type of immunobiological. Goiás State, Brazil, August 2014 to December 2017

Immunobiologicals	IE*	Doses	IR^†^	95% CI^‡^
**Routine**				
Triple Viral	77	880,192	8.74	7.00-10.93
Yellow Fever	60	961,916	6.34	4.85-8.03
Human Papillomavirus	50	493,767	10.12	7.68-13.35
Pentavalent	37	1,186,555	3.12	2.26-4.30
Oral Human Rotavirus Vaccine	35	598,092	5.85	4.20-8.14
Conjugated meningococcal C	31	2,120,077	1.46	1.03-2.08
Triple Bacterial	28	507,584	5.52	3.82-7.97
Oral Polio Vaccine	27	480,871	5.61	3.86-8.20
Hepatitis B	25	972,990	2.57	1.74-3.79
Pneumococcal 10 valent	18	995,218	1.81	1.14-2.86
Adult Double	14	1,353,737	1.03	0.62-1.74
Inactivated Polio Vaccine	13	695,891	1.87	1.09-3.20
*Bacillus Calmette-Guérin*	12	326,151	3.68	2.10-6.43
Tetra Vital	12	223,103	5.38	3.01-9.40
Human rabies vaccine	10	40,109	24.93	13.54-45.89
Hepatitis A (pediatric)	7	288,564	2.43	1.17-5.00
Triple Acellular Bacterial Adult	8	149,893	5.34	2.70-10.53
**Subtotal**	**464**	**12,274,710**	**3.78**	**3.45-4.14**
**Serums**				
Scorpion serum	5	544	919.12	393.20-2133.00
Serum against tetanus	1	385	259.74	45.87-1456.00
Botropic serum	1	349	286.50	50.60-1605.0
**Subtotal**	**7**	**1,278**	**547.70**	**265.6-1126.0**
**Special immunobiologicals**				
Pneumococcal 23	8	3,299	242.50	122.90-477.80
Hepatitis A (CRIE^§^)	2	2,719	73.56	20.18-267.80
Triple Acellular Bacterial Infant	2	2,856	70.03	19.21-255.00
Vaccine *Haemophilus influenzae* type b	1	1,275	78.43	13.85-442.90
Immunoglobulin anti-hepatitis B	1	1,820	59.94	9.70-310.6
**Subtotal**	**14**	**11,969**	**116.97**	**69.69-196.30**
**Campaign**				
Trivalent Influenza	16	74,341	21.52	13.25-34.96
**Total**	**501**	**12,362,298**	**4.05**	**3.71-4.42**

Source: Information System of the National Immunization Program (IS-PNI), IS-AEFI module.

* IE = Immunization error;

† IR = Incidence rate: every 100,000 applied doses;

‡ CI 95% = 95% Confidence Interval;

§ CRIE = Reference Center for Special Immunobiologicals

Tables 4 and 5 show the incidence rate of IE without AEFI and with AEFI, in Goiás (per 100,000 a.d.), respectively.

**Table 4:** Incidence rate of immunization errors without Post-Vaccination Adverse Event (*per* 100,000 applied doses), according to vaccination strategy and type of immunobiological. Goiás State, Brazil, August 2014 to December 2017

Immunobiologicals	IE*	Doses	IR^†^	95% CI^‡^
**Routine**				
Triple Viral	74	880,192	8.40	6.65-10.49
Yellow Fever	56	961,916	5.82	4.44-7.50
Human Papillomavirus	46	493,767	9.32	6.90-12.32
Oral Human Rotavirus Vaccine	34	598,092	5.68	4.00-7.85
Pentavalent	32	1,186,555	2.70	1.88-3.76
Conjugated meningococcal C	30	2,120,077	1.41	0.97-1.99
Oral Polio Vaccine	27	480,871	5.61	3.86-8.20
Hepatitis B	25	972,990	2.57	1.74-3.79
Triple Bacterial	25	507,584	4.92	3.26-7.16
Pneumococcal 10 valent	17	995,218	1.71	1.03-2.68
Tetra Viral	11	223,103	4.93	2.59-8.57
Inactivated Polio Vaccine	11	695,891	1.58	0.83-2.75
Adult Double	10	1,353,737	0.74	0.37-1.32
Human rabies vaccine	9	40,109	22.44	10.94-41.18
Triple Acellular Bacterial Adult	8	149,893	5.34	2.70-10.53
Hepatitis A (pediatric)	7	288,564	2.43	1.17-5.00
*Bacillus Calmette-Guérin*	1	326,151	0.31	0.01-1.51
**Subtotal**	**423**	**12,274,710**	**3.45**	**3.13-3.82**
**Serums**				
Scorpion serum	3	544	551.50	140.30-1,501.00
Serum against tetanus	1	385	259.74	45.87-1,456.00
Botropic serum	-	-	-	-
**Subtotal**	**4**	**1278**	**313.00**	**84.21-801.03**
**Special immunobiologicals**				
Pneumococcal 23	6	3,299	181.90	73.72-378.30
Hepatitis A (CRIE^§^)	2	2,719	73.56	20.18-267.80
Triple Acellular Bacterial Infant	2	2,856	70.03	19.21-255.00
Vaccine *Haemophilus influenzae* type b	1	1,275	78.43	13.85-442.90
Immunoglobulin anti-hepatitis B	1	1,820	59.94	9.70-310.6
**Subtotal**	**12**	**11,969**	**100.30**	**51.75-175.10**
**Campaign**				
Trivalent Influenza	15	74,341	20.18	11.29-33.28
**Total**	**454**	**12,362,298**	**3.42**	**3.11-3.76**

Source: Information System of the National Immunization Program (IS-PNI), IS-AEFI module.

* IE = Immunization error;

† IR = Incidence rate: every 100,000 applied doses;

‡ CI 95% = 95% Confidence Interval;

§ CRIE = Reference Center for Special Immunobiologicals

**Table 5:** Incidence rate of immunization errors with Adverse Post-Vaccination Event (per 100,000 applied doses), according to vaccination strategy and type of immunobiological. Goiás State, Brazil, August 2014 to December 2017

Immunobiologicals	IE*	Doses	IR^†^	95% CI^‡^
**Routine**				
Triple Viral	3	880,192	0.34	0.12-1.00
Yellow Fever	4	961,916	0.41	0.16-1.07
Human Papillomavirus	4	493,767	0.81	0.31-2.08
Oral Human Rotavirus Vaccine	1	598,092	0.17	0.03-0.95
Pentavalent	5	1,186,555	0.42	0.18-0.99
Conjugated meningococcal C	1	2,120,077	0.05	0.00-0.27
Triple Bacterial	3	507,584	0.59	0.20-1.74
Pneumococcal 10 valent	1	995,218	0.10	0.02-0.57
Tetra Vital	1	223,103	0.45	0.08-2.54
Inactivated Polio Vaccine	2	695,891	0.29	0.08-1.05
Adult Double	4	1,353,737	0.29	0.11-0.76
Human rabies vaccine	1	40,109	2.49	0.44-14.12
*Bacillus Calmette-Guérin*	11	326,151	3.37	1.88-6.04
**Subtotal**	**41**	**10,382,392**	**0.39**	**0.29-0.54**
**Serums**				
Scorpion serum	2	544	367.65	100.90-1,330.00
Botropic serum	1	349	286.53	50.60-1,605.00
**Subtotal**	**3**	**893**	**335.95**	**114.30-983.00**
**Special immunobiologicals**				
Pneumococcal 23	2	3,299	60.62	16.63-220.80
**Subtotal**	**2**	**3,299**	**60.62**	**16.63-220.80**
**Campaign**				
Trivalent Influenza	1	74,341	1.34	0.23-7.62
**Total**	**47**	**10,460,925**	**0.45**	**0.34-0.60**

Source: Information System of the National Immunization Program (IS-PNI), IS-AEFI module.

* IE = Immunization error;

† IR = Incidence rate: every 100,000 applied doses;

‡ CI = 95% Confidence Interval 95%

The IE incidence rate without AEFI was 3.42 IE/100,000 a.d. Vaccines administered routinely had an incidence rate of 3.45/100,000 a.d. and serums of 313.00 IE/100,000 a.d. The three highest IE rates incidence without AEFI for routine vaccines were found for the human rabies vaccine (22.44 IE/100,000 a.d.), human papilloma virus (9.32/100,000 a.d.) and triple viral (8.40/100,000 a.d.) (Table 4).

The IE incidence rate with AEFI was 0.45 IE/100,000 a.d. Routine vaccines had an incidence rate of 0.39 IE/100,000 a.d. and serums of 335.95 IE/100,000 a.d. (Table 5).

Of the total IE with AEFI analyzed (n = 47), there were 139 different AEFI, including local manifestations (92; 66.2%) and systemic (47; 33.8%). The five most reported AEFI were: local pain (20; 14.4%), edema or flushing (17; 12.2%), erythema (17; 12.2%), heat (13; 9.4%) and nodule (7; 5,0%). Regarding the type of IE with AEFI, the error in the administration technique was the most frequent (22; 46.8%), followed by the inadequate interval between doses (10; 21.3%), type of immunobiological used (6; 12.8%), evaluation of contraindications or precautions (5; 10.6%), prescription or indication errors (2; 4.3%) and others (2; 4.3%) (data not shown in tables).

## Discussion

In the public health, vaccination is a safe strategy capable for significantly impacting the control or elimination of preventable diseases^(^ 21 ^-^ 22 ^)^. In this sense, safe vaccination is a worldwide concern and a determining factor in the immunization programs success or failure^(^ 23 ^-^ 24 ^)^. In this study, 501 IE were analyzed, the majority involving routine doses (92.6%), without AEFI (90.6%) and in children under five years old (55.7%). The most frequent types of errors pointed out the inadequacy in the indication of the immunobiological (26.9%), inadequate interval between doses (18.2%) and error in the administration technique (14.2%). The overall incidence rate for IE was 4.05/100,000 a.d.; the highest incidences of routine vaccines were for human rabies vaccine, human papillomavirus and triple viral; the incidence rate of errors with AEFI was 0.45/100.00 a.d..

The study’s limitations come from the analysis of data coming from secondary reporting systems, prone to certain biases, including the absence of information, underreporting of incidents and the IE. These biases may underestimate IE incidence rate. In this investigation, notifications were identified with a lack of information for certain variables, such as the route of administration and race/skin color. In addition, differences in the reporting profile may have occurred between reporting institutions, since those with a well-established safety culture are likely to contribute to more complete notifications and information than those whose punitive culture still prevails^(^ 9 ^,^ 25 ^)^. Thus, the results of this study may not represent IE real magnitude for the analyzed location.

The quality of immunization programs depends on how vaccines are produced, transported, packaged, prepared and administered^(^ 26 ^)^, as well as the quality of the notifications and the analysis of the occurred IE, one of the great challenges for the immunization services is to certify safe vaccination practices. The analysis of events reported to the USA National Vaccine Adverse Event Reporting System shows that IE remain present in the clinical practice^(^ 3 ^,^ 25 ^)^. This study confirms this reality and points out that the IE are found in the Brazilian reality, being more frequent in children under five years old, especially in children under one year old.

The children under one year old have been hardest hit by AEFI^(^ 15 ^,^ 18 ^)^and by IE with AEFI^(^ 6 ^)^. This fact may be due to the greater vaccine exposure to which this age group is exposed, considering that the majority of vaccines that make up the calendar are indicated for children under one year old^(^ 14 ^)^.

In this study, proportionally, the majority of IE occurred with immunobiologicals administered intramuscularly, followed by those administered subcutaneously. In Brazil, most routine vaccines are composed of those administered intramuscularly, in addition to special immunobiologicals and serums^(^ 14 ^)^, a fact that may explain the result of this study.

In this investigation, it was found that the IE majority occurred during routine vaccination. Also, a higher proportion of IE was observed related to triple viral vaccines, yellow fever, HPV, pentavalent and OVHR. These findings corroborate a study carried out in the Southeast Region of Brazil, in which there was also a higher proportion of IE related to routine vaccines (79.0%) and human rotavirus vaccines (22.0%), fever yellow (15.6%) and tetravalent (12.9%)^(^ 17 ^)^. In another study carried out in the Midwest Region of Brazil, AEFI notification forms for children under five years of age were analyzed, among them, the majority of IE involved the administration of yellow fever and OPV vaccines^(^ 18 ^)^. When comparing with international literature, an investigation conducted in the USA showed that HPV(26.0%) and rotavirus (15.0%) vaccines were the most involved in the IE in the clinical practice^(^ 3 ^)^.

In this study, it was found that the most prevalent types of IE were failures in prescription and/or indication of the immunobiological, followed by the inadequate interval between doses and error in the administration technique. A research that analyzed IE reported in Europe, from 2001 to 2016, showed that the most frequent IE categories included incomplete vaccination (36.1%), errors in administration (22.1%), administration of the wrong vaccine or at the wrong age (14.6%)^(^ 8 ^)^. In a study conducted through the analysis of notifications from a population-based immunization service in the United Kingdom, it was found that 92%of errors occurred during the selection and preparation of the vaccine^(^ 26 ^)^. In the USA, a study carried out from 2000 to 2013 showed that the most common IE groups indicated the use of an inappropriate scheme (27.0%), errors in the storage and dispensing of immunobiologicals (23.0%) and incorrect vaccine (15.0%)^(^ 3 ^)^. Other studies have pointed out that errors that occur in the administration stage are the most common^(^ 3 ^,^ 9 ^,^ 25 ^-^ 26 ^)^and included wrong number of doses, inadequate dose interval and wrong vaccine^(^ 9 ^)^.

Of the total analyzed IE, 9.4% were IE with AEFI. The IE IT with AEFI was 0.45 IE/100,000 a.d. Similar results have been identified in other studies conducted in Brazil^(^ 6 ^,^ 15 ^,^ 18 ^)^. The results reported in the international literature reveal divergent data regarding the magnitude of the AEFI followed by IE. A report drawn up from an Australian database revealed 2,924 AEFI related to IE cases in 2015, which represented 12.3 events for every 100,000 vaccinated people^(^ 27 ^)^. In Canada, a study on 3,504 AEFI reports estimated an IE rate of 0.39 per 100,000 a.d.^(^ 11 ^)^. In the USA, an analysis of the AEFI surveillance system showed that in the period from 2000 to 2013, 21,843 IE cases were reported in the country and that, in 25% of the cases, there was some harm to the user^(^ 3 ^)^.

In addition, the analysis of IE with AEFI showed the occurrence, mainly, of local manifestations, such as pain, edema or flushing, erythema, heat and nodules. Other studies conducted in Brazil identified that more than half of the manifestations related to the AEFI reported were local and self-limited, spontaneously resolved and without the need for medical intervention^(^ 15 ^,^ 18 ^)^. On the other hand, a study conducted in a state in the Southern Region showed that AEFI followed by more frequent IE showed hot subcutaneous abscess, cold subcutaneous abscess and suppurated regional lymphadenopathy^(^ 6 ^)^. A Canadian study found that the majority of IE with AEFI were mild or of moderate severity, indicating that the commonly reported reactions included extensive, prolonged or painful local reactions in a member of the injection site^(^ 28 ^)^.

It is worth considering that severe IE-related AEFI are rare, so the benefits of immunization are significantly greater than the problems that may eventually occur^(^ 29 ^)^. However, many of these events can be prevented if health services establish effective infrastructure to monitor and promote safe practices, including the release of annual reports^(^ 30 ^)^.

Regarding the vaccines most involved in IE with AEFI, *Bacillus Calmette-Guérin* (BCG) and the pentavalent were the most common. BCG intradermal vaccination is associated with specific errors^(^ 31 ^)^ which can cause adverse events ranging from local manifestations to more severe manifestations^(^ 32 ^)^.

Although most IE do no harm to users, efforts should be directed towards planning actions aimed at improving the quality of care provided to the population^(^ 2 ^-^ 3 ^)^. Monitoring the AEFI, particularly those caused by IE, is important for the success of immunization programs, as such events can influence the acceptance of immunization by the population, as well as negatively impact costs for the health system^(^ 3 ^)^. Therefore, IE epidemiological assessment may come to contribute to the changes in practice, directing efforts to reduce errors and harm caused to users, as well as reducing costs incurred in the AEFI treatment.

It is highlighted that, with the inclusion of new immunobiologicals^(^ 12 ^)^, the profile of the vaccination calendar in Brazil has changed considerably in recent years. Complexity of immunization makes the work of health managers and professionals challenging to guarantee safety in the vaccination process. Managers and professionals must continually pay attention to the fact that the production of safe and effective vaccines is not enough, but also to ensure adequate structures and processes that favor the safe development of all stages that make up the immunization system^(^ 33 ^)^.

Vaccination is an action that has been carried out by nursing and, in Brazil, more frequently by nursing technicians, considering that nurses accumulate other activities, both managerial and assistance related^(^ 34 ^)^. Ensuring the skills and updates for this practice, adopting systemic measures that help them not to make mistakes, is essential to increase safety in this process.

The services must guarantee the constant presence of the nurse within the vaccination rooms and, mainly, in the screening, developing nursing consultation with a view to the adequate assessment and indication of the immunization. It should be noted that the PNI recommends that, for the dimensioning of nursing staff, it should be based on the fact that a vaccinator can safely administer about 30 doses of injectable vaccines or 90 doses of vaccines administered by oral route per hour of work^(^ 14 ^)^. This assertion disregards the complexity of the process and the time spent by the nursing professional to carry out all the necessary actions for safe immunization, such as screening; user rating; hand hygiene (before preparation, before and after administration of doses); the preparation of doses, often needing reconstitution; the conference, the records and the guidance to users, implying insufficient human resources and overload for the nursing professional. The deficit of professionals and the consequent work overload are causes of errors and incidents caused by nursing, which have a close relationship with the management of health services^(^ 35 ^)^.

They are still incipient, but potential strategies aimed at reducing the IE are being suggested, including: ensuring the professional competence of the teams, particularly nursing professionals, regarding the correct technique for administering immunobiologicals; educate professionals and users of the system about the interval between doses, especially those with complex administration regimes; ensure well-defined processes for the safe administration of vaccines and other immunobiologicals; and investigate the causes for all IE in order to prevent future occurrences^(^ 3 ^,^ 33 ^)^.

Other strategies for preventing IE concern using checklists^(^ 31 ^)^; investments in information technology infrastructure to support clinical decision-making; aid in identifying IE risk factors^(^ 9 ^)^ and permanent education in health for all professionals involved with immunization in the country’s health services. Such strategies can be considered as transforming practices aimed at the safety of the users^(^ 36 ^-^ 37 ^)^.

However, it is worth mentioning that the educational activities carried out in the permanent education in health actions must be planned based on the problematization of daily life and the analysis of care failures so that they generate reflections, improvement actions and serve as learning for all^(^ 36 ^)^. Teaching strategies with active methodologies, realistic simulation and discussion of errors that occurred are also important to bring students and professionals closer to the reality to be faced^(^ 34 ^)^.

In addition to permanent education in health, it is essential to develop skills for immunization safety in undergraduate courses for health professionals, with an emphasis on nurses, responsible for vaccination rooms^(^ 34 ^)^.

The users of the system also need to be involved in the immunization process, so that they are able to not only serve as a barrier to a possible error, but also to detect and report signs of adverse events^(^ 27 ^)^, having as one of the possible strategies the verification by the user of their own data, the child under his responsibility and the immunological one before its preparation and administration.

Careful surveillance and IE investigation are necessary to identify the causes of these events that require correction^(^ 38 ^)^. Effective spontaneous reporting of the IE is the first step in ensuring that vaccines are safe and that they are being administered correctly^(^ 39 ^)^.

## Conclusion

Vaccination has brought immeasurable benefits to collective health, significantly reducing, controlling and eradicating preventable diseases. Thus, in order to ensure your success it is important that failures do not occur during the process. However, the evidences from that study indicate that the immunization errors are a reality to be faced by health systems and nursing, impacting the quality of care and the safety of users who seek to prevent the illness in the population.

Most IE occurred in children under the age of five, it did not cause harm and the incidence rate found was low and close to the few studies carried out, nationally and internationally. However, when it comes to lives, actions to prevent and minimize these occurrences must be adopted, focused on analyzing the structure and processes of immunization.

This study carried out an analysis of immunization errors, which can subsidize management in decision-making towards strengthening the quality of vaccination procedures. Thus, it fulfills the role of disseminating information on issues related to the safety of users of the health system in the context of immunization and amplifying the access of management, professionals and the population on this theme.

The study also has implications on the care practice and for the teaching in the health and nursing field, as it displays situations that alert for a closer approach to nurses in the vaccination room, aiming to improve supervision, permanent education in health, risk management and the direct assistance to the system users. Using protocols, tools and dynamics with a systemic and non-individual view is also fundamental, not only to mitigate errors, but also to maintain the confidence, quality and positive impact of the PNI, at all instances.
